# NIRSLINK: A Modular Cascaded Wearable Near-Infrared Spectroscopy System for High-Speed Multi-Site Hemodynamic Monitoring

**DOI:** 10.3390/bios16090472

**Published:** 2026-08-28

**Authors:** Shuo Zhang, Kangkang Xu, Nan Zeng, Jiansong Sun, Qianrui Yang, Qianke Zeng, Zheng Ding, Yanyu Lu, Jian Zhao, Mohamad Sawan, Shan Fu, Guoxing Wang, Cheng Chen

**Affiliations:** 1 Department of Micro/Nano Electronics, Shanghai Jiao Tong University, Shanghai 200240, China; 2 School of Automation and Intelligent Sensing, Shanghai Jiao Tong University, Shanghai 200240, China; 3 CenBRAIN Neurotech, Westlake University, Hangzhou 310030, China

**Keywords:** near-infrared spectroscopy (NIRS), wearable sensor, modular design, flexible probe, cascaded architecture, multi-site

## Abstract

Wearable near-infrared spectroscopy (NIRS) enables non-invasive hemodynamic monitoring, yet conventional CW-NIRS systems suffer from limited temporal resolution, scalp-only measurement, and mandatory manual tuning to compensate for optical heterogeneity across subjects and sites. This work develops NIRSLINK, a modular cascaded wearable NIRS system for high-speed multi-site hemodynamic acquisition. Its flexible probes adopt spring-floating optics with a standardized 30 mm optode separation, integrating dual 735/850 nm LEDs, silicon photodiodes, and a two-stage closed-loop tuning algorithm. A single probe achieves a peak sampling rate of 3 kHz, and up to eight cascaded probes form 52 valid channels, with a signal-to-noise ratio (SNR) of 78.61 ± 7.03 dB and an optical dynamic range (DR) of 101.32 ± 12.41 dB. Phantom experiments verify its millisecond temporal resolution and high sensitivity to blood flow and hemoglobin variations. In vivo trials, including the Valsalva maneuver, forearm occlusion, and two-back cognitive tasks, demonstrate simultaneous recording of hemoglobin concentration shifts, pulse waveforms, and beat-to-beat pulse transit times (PTTs). NIRSLINK supports hemodynamic measurements across multiple anatomical locations, including the forehead, forearm, upper arm, and thigh, covering both cranial and peripheral body regions. The embedded auto-tuning module stabilizes signals from the forehead, forearm, and other regions within the optimal ADC range without manual adjustment, preventing signal saturation and SNR degradation. This scalable adaptive platform overcomes critical drawbacks of traditional wearable NIRS, applicable to cognitive neuroscience, non-invasive cardiovascular assessment, and ambulatory physiological monitoring, and provides design references for multi-site optical sensors.

## 1. Introduction

Near-infrared spectroscopy (NIRS) has become an established non-invasive optical modality for probing tissue hemodynamics, with applications ranging from cortical functional mapping to peripheral muscle oxygenation assessment [1,2,3]. The absence of ionizing radiation, portability, and relatively low implementation costs have facilitated its transition from laboratory-grade neuroimaging tools to wearable sensors for ambulatory health monitoring [4,5,6]. Among these, continuous-wave NIRS (CW-NIRS) systems are particularly favored for wearable applications due to their simpler hardware architecture and capability for real-time tracking of tissue blood oxygenation changes. Despite these developments, conventional wearable CW-NIRS platforms continue to face several limitations that impede their broader adoption in comprehensive physiological monitoring [7].

Three technical challenges remain inadequately addressed in wearable NIRS. First, temporal resolution often decreases in multichannel configurations; many systems provide effective sampling rates of only 10–50 Hz when multiple channels are active [8]. Centralized analog-to-digital converter architectures and sequential channel scanning introduce multiplexing delays that hinder extraction of high-frequency physiological features. Second, most modular systems are designed for cranial monitoring, with probe geometry, rigidity, and optical coupling optimized for the scalp. This cranial focus limits their use on the forearm, carotid artery, and lower limbs, where curvature, elasticity, and optical properties differ from those of the scalp [9]. Flexible probes that maintain stable contact across anatomical surfaces are needed for systemic vascular assessment and multi-site hemodynamic connectivity studies. Third, between-participant and between-site variations in melanin content, adipose-layer thickness, and scattering properties require adjustments of gain and light intensity to avoid saturation or poor SNR. Manual tuning is impractical for adaptive deployment and longitudinal self-monitoring [10], whereas fixed-gain architectures cannot accommodate this optical heterogeneity and may compromise derived physiological parameters [11].

Previous systems have addressed subsets of these limitations. Zhao et al. developed a mechanically flexible, high-density diffuse optical tomography (DOT) system for neonatal brain monitoring that improved scalp conformability, but it was designed for cranial rather than peripheral measurements [12]. Wyser et al. reported a modular multiwavelength NIRS device with short-separation channels for superficial signal correction; its reported maximum sampling rate was 100 Hz [13]. Ban et al. developed a high-channel-count time-domain NIRS (TD-NIRS) platform with improved temporal sampling, but its fiber-coupled detectors and optomechanical components were designed for cranial neuroimaging rather than peripheral wearable monitoring [14]. More recent platforms have incorporated front-end digitization and cascaded topologies to reduce fiber-related artifacts, yet they do not concurrently address high-speed multi-site acquisition and participant-adaptive tuning [15,16,17,18,19].

To address these gaps, we present NIRSLINK, a modular cascaded wearable NIRS system designed for high-speed multi-site hemodynamic monitoring with integrated adaptive signal conditioning. The system is characterized by three technical features. Through a distributed front-end digitization architecture and a time-division data transmission scheme, NIRSLINK achieves a sampling rate of up to 3 kHz for a single probe (4 channels) when connected. The lightweight flexible probes incorporate a spring-loaded floating optical coupling mechanism that conforms to body surfaces with varying curvatures, including the forehead, forearm, and carotid artery, maintaining stable skin contact and optical coupling across different sites, thereby supporting simultaneous monitoring of cerebral, peripheral arterial, and muscular hemodynamics. An adaptive two-stage closed-loop tuning algorithm, which employs a successive approximation algorithm, jointly adjusts the programmable gain amplifier (PGA) and transimpedance amplifier (TIA) gain settings and light-emitting diode (LED) drive current to tune the acquired value to a preset target. This real-time adaptive tuning mechanism eliminates the need for manual gain configuration, compensates for inter-individual and inter-site variations in optical properties, and maintains the acquired signals within the optimal input range of the analog-to-digital converter (ADC), thereby preserving a high SNR across a wide dynamic range while preventing saturation.

We evaluated system performance through phantom experiments and human subject experiments, including the Valsalva maneuver, forearm vascular occlusion, a two-back working memory task, carotid–radial pulse wave velocity (PWV) estimation, and a motion feasibility protocol. Experimental results demonstrate that NIRSLINK captures millisecond-scale transient events and records physiologically meaningful hemodynamic responses at both cerebral and peripheral sites, with consistent signal quality across subjects without manual tuning. Collectively, these results establish NIRSLINK as a scalable and adaptable wearable platform for multi-site hemodynamic monitoring, with potential utility in cognitive neuroscience, cardiovascular function assessment, and ambulatory health applications.

## 2. Materials and Methods

### 2.1. System Description

**Figure 1:**
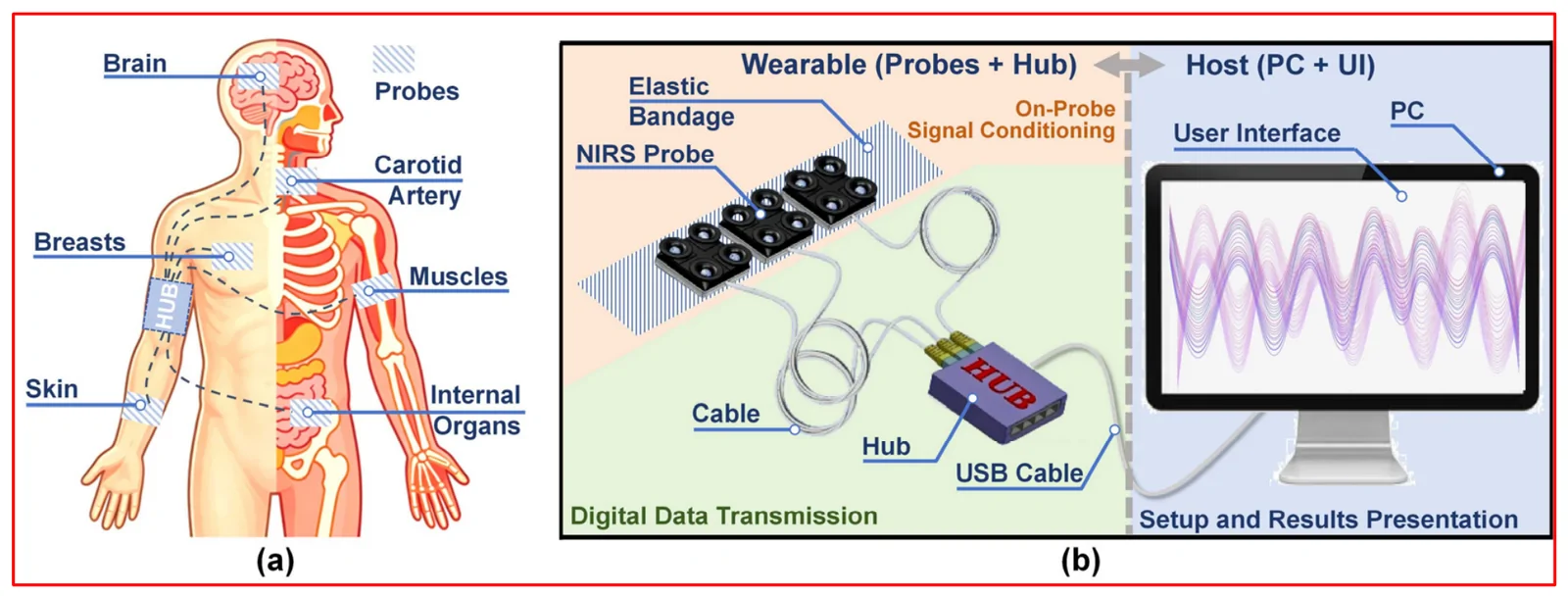
Schematic overview of NIRSLINK: (**a**) Probe deployment across multiple sites (brain, carotid artery, muscle, and skin) for multi-site monitoring. (**b**) System architecture comprising cascaded NIRS probes, a central hub, and a host computer. This configuration highlights the system’s capacity to scale from localized sensing to synchronized multi-site hemodynamic monitoring within a unified acquisition architecture.

As shown in Figure 1, NIRSLINK consists of cascaded NIRS probes, a central hub, and a host computer. The cascaded probes serve as the fundamental sensing units and support flexible spatial reconfiguration and expansion. The number of probes can be adjusted according to the monitoring region and coverage requirements, allowing the system to form a scalable sensor array for hemodynamic acquisition at multiple sites, including the scalp, forehead, forearm, and carotid artery.

Each probe connects to the hub through lightweight flexible cables up to 100 cm long. The miniaturized hub can be placed on any part of the body, such as the upper arm, allowing the wearer to move within a prescribed range while still enabling continuous monitoring. Each integrated probe contains dual-wavelength LEDs and dual photodiodes (PDs). A cable provides both power supply and data communication, simplifying the wearable wiring layout. Unlike conventional NIRS devices that transmit analog optical signals through fibers, NIRSLINK performs in situ optoelectronic conversion and signal digitization at each front-end probe; only digital data travel through the connecting cables. This architecture reduces wearable burden, suppresses cable-induced interference and motion artifacts, and improves signal stability during dynamic monitoring.

### 2.2. Hardware Design

#### 2.2.1. Optical Unit

**Figure 2:**
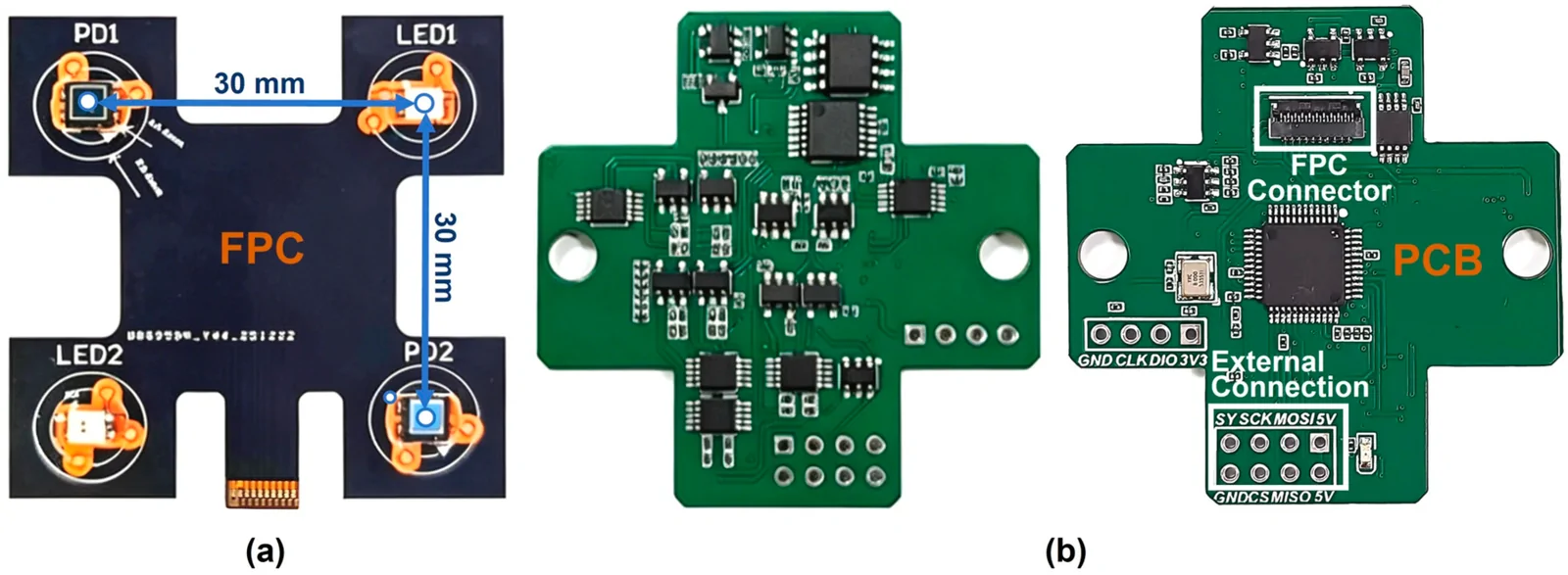
Photo of the fabricated NIRS probe circuit board: (**a**) FPC carrying dual-wavelength LEDs and PDs with a source-detector spacing (SDS) of 30 mm. (**b**) Front and rear views of the rigid PCB that houses the signal processing. The compact separation of the conformable optical interface from the rigid processing board enables stable optode placement while retaining on-probe signal conditioning.

**Figure 5:**
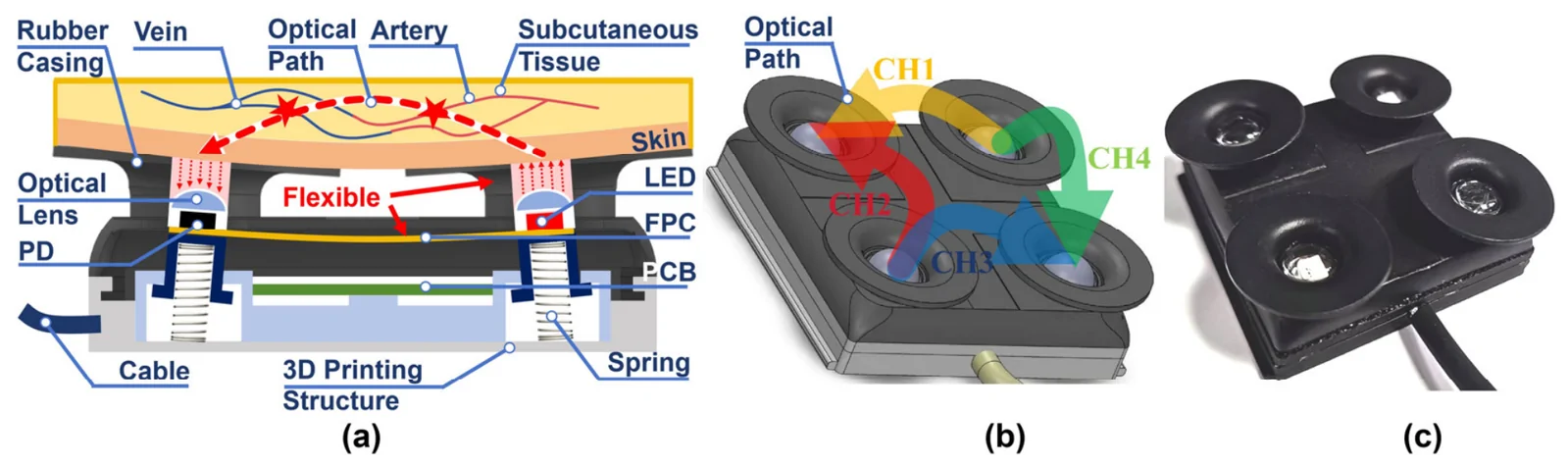
Structural and packaging design of the wearable probe: (**a**) Cross-sectional view showing the flexible optical unit, spring-loaded mechanism, rigid electronic module, and skin interface; the tissue optical path is indicated. (**b**) Optical channel layout within each probe. (**c**) Photograph of the assembled probe. The mechanically decoupled, spring-loaded optical interface improves conformity to curved anatomical surfaces, which is essential for maintaining optical coupling during multi-site wearable measurements.

The optical sensing module in each NIRS probe, as shown in Figure 2, uses surface-mount optoelectronic devices. A dual-wavelength LED (SMT735D/850D, USAUSHIO Opto Semiconductors, Kyoto, Japan), with emission peaks at 735 nm and 850 nm, provides near-infrared illumination for tissue measurement. A silicon PD (S12158-01CT, Hamamatsu Photonics K.K., Hamamatsu, Japan) serves as the optical detector. All optoelectronic components are mounted on a flexible printed circuit (FPC) that bends freely to accommodate the curvature of the skin. Dedicated connectors link the FPC to the rigid printed circuit board (PCB), ensuring reliable power delivery and signal transmission. Notably, the rigid PCB only accommodates back-end signal processing circuits and is mechanically decoupled from the front FPC optical assembly via a spring-loaded floating structure, shown in Figure 5a. The FPC carrying optoelectronic devices forms the deformable front layer facing human skin and provides intrinsic bending compliance for curved tissue surfaces. The front FPC assembly can undergo independent three-dimensional bending without mechanical constraints from the rigid PCB, enabling stable conformal optical coupling on uneven anatomical regions.

#### 2.2.2. Circuit Unit

**Figure 3:**
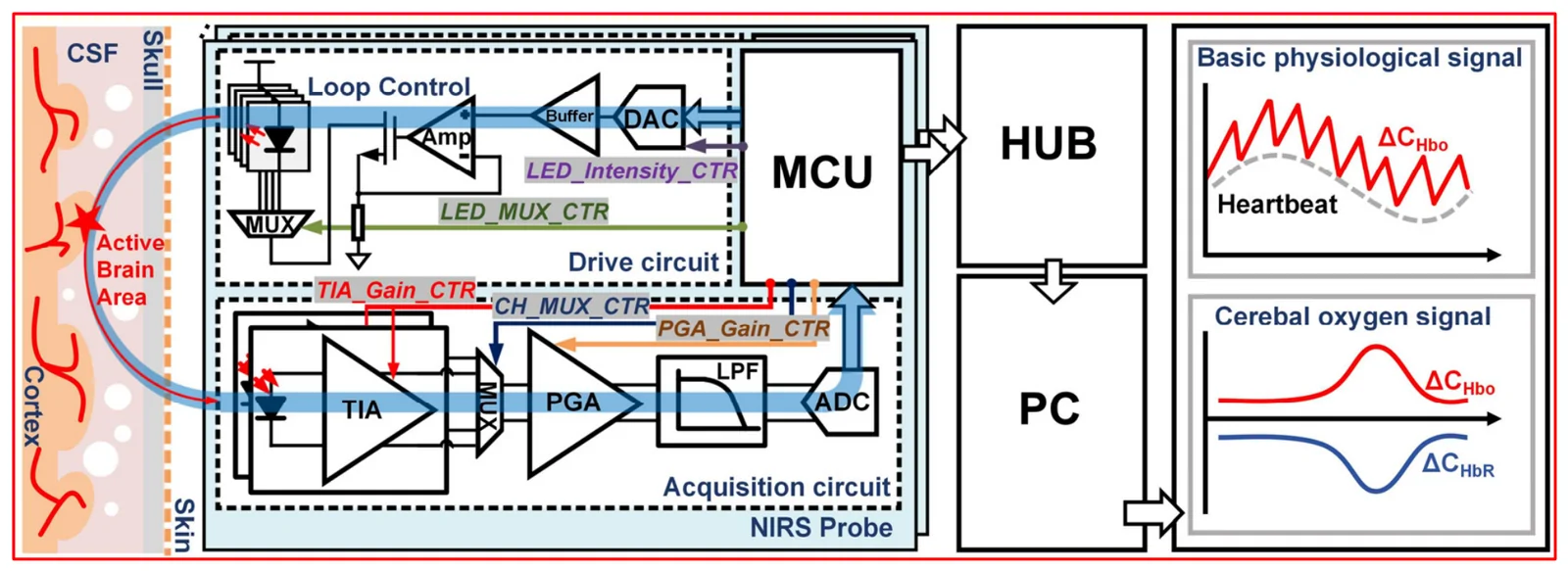
Block diagram of the NIRS probe system. The left portion illustrates the optical path through skin, skull, and cerebrospinal fluid (CSF) to the cerebral cortex. Each probe integrates a drive circuit and an acquisition circuit; digitized data are transmitted via the hub to the host computer for extraction of physiological signals, including cardiac pulsations and cerebral oxygenation. Local signal conditioning and digitization shorten the analog signal path, thereby reducing cable-induced interference and supporting reliable extraction of both slow hemodynamic changes and fast pulsatile components.

The circuit unit is the core processing module of each NIRS probe. It generates stable drive signals for the optical unit, conditions and digitizes the acquired photoelectric signals, and supports bidirectional communication with the central hub. Together with the flexible front-end optical sensing module, it forms a complete signal acquisition chain for wearable NIRS monitoring. Figure 3 shows the overall hardware schematic of the circuit unit.

The circuit unit comprises an LED drive circuit and a signal acquisition circuit. The drive circuit provides high-precision constant-current excitation for the dual-wavelength LEDs. The microcontroller unit (MCU, CH32F103C8T6, Nanjing Qinheng Microelectronics Co., Ltd., Nanjing, China) outputs light-intensity control signals (LED_Intensity_CTR), which are converted by a digital-to-analog converter (DAC, AD5551, Analog Devices Inc., Wilmington, MA, USA) and subsequent current driver stages into adjustable and stable drive currents. A high-speed analog multiplexer (ADG1404, Analog Devices Inc., Wilmington, MA, USA) supports time-division multiplexing for multichannel LED control (LED_MUX_CTR).

The acquisition circuit conditions the weak photocurrents generated by the PDs. Raw nanoampere-level photocurrents first enter low-noise TIAs, which convert current to voltage with high precision. Two selectable transimpedance gains, 1 MΩ and 10 MΩ, provide adjustable amplification (TIA_Gain_CTR). Because the optoelectronic signals are extremely weak, the TIA stage must provide both low noise and stable operation. Time-division multiplexing can introduce switching noise and parasitic interference; therefore, each detection channel uses an independent TIA to reduce crosstalk and noise coupling [20].

Multiplexers controlled by the MCU (CH_MUX_CTR) sequentially gate the amplified TIA outputs and route them to the PGA for secondary amplification. The PGA offers two selectable gains, 1 and 5, for further amplitude adjustment (PGA_Gain_CTR). The two-stage amplification architecture (TIA + PGA) therefore enables flexible gain configuration while limiting circuit volume, power consumption, and hardware redundancy. The conditioned signals then pass through a low-pass filter to suppress power-line interference and high-frequency random noise before digitization by a high-resolution analog-to-digital converter (AD7982, Analog Devices Inc., Wilmington, MA, USA).

An MCU serves as the timing and control core of the circuit. It coordinates LED illumination timing, gain configuration, and ADC sampling, while closed-loop feedback enables full parameter optimization. The MCU transmits the digitized data to the central hub through a 4 MHz serial peripheral interface, ensuring efficient and stable data transfer. The hub then uploads the integrated data to the host computer through a universal serial bus (USB) interface. This hierarchical communication architecture provides reliable end-to-end data transmission for high-precision, long-term physiological signal acquisition.

### 2.3. Software Design and Cascading Scheme

#### 2.3.1. Timing Control and Working Mechanism of Multi-Probe Cascading

**Figure 4:**
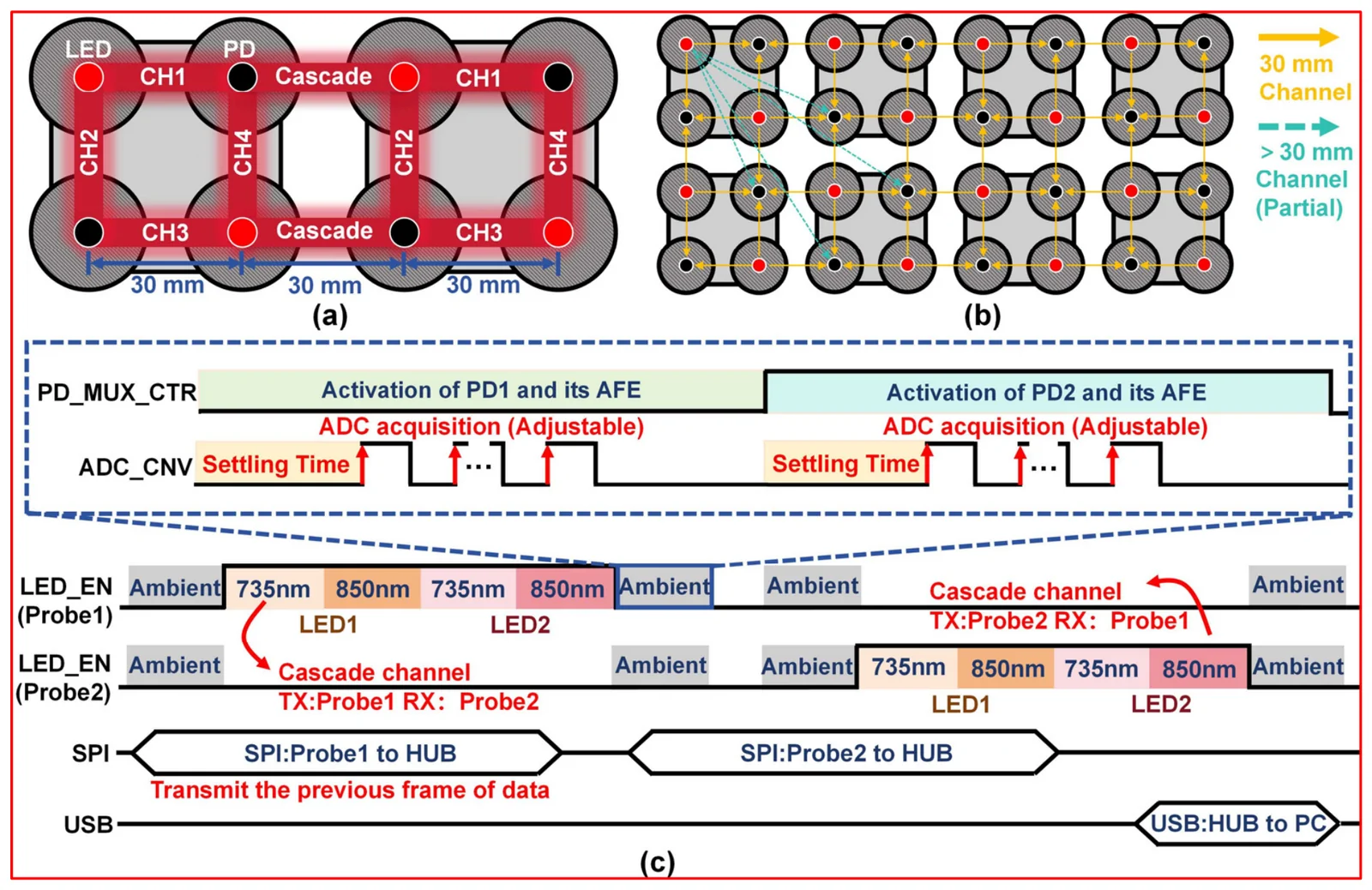
Probe array configuration and timing diagram: (**a**) Formation of intra- and inter-probe detection channels with uniform 30 mm spacing. (**b**) Valid and invalid channel distributions for an eight-probe cascaded array. (**c**) Timing control for PD gating, ADC sampling, light source activation, and probe data transmission. The combination of uniform channel geometry and overlapped acquisition/transmission timing enables scalable spatial coverage while preserving synchronized operation across the cascaded array.

Under onboard MCU control, the acquisition workflow follows the timing scheme shown in Figure 4c. After acquisition is triggered, the dual-wavelength LEDs emit near-infrared light sequentially. Because wearable in vivo optical measurements are sensitive to ambient stray light, the system samples background signals before and after each LED illumination phase. A software algorithm subtracts this background component from the raw optical data, thereby suppressing ambient noise and improving the SNR of the hemodynamic signals.

All PDs begin acquisition after a fixed delay following LED activation. This delayed sampling strategy avoids transient jitter caused by instantaneous switching. The system features configurable sampling point counts and averaging numbers within each sampling cycle, enabling adjustable sampling rates. By varying the averaging count, a direct trade-off is established between the sampling rate and the attenuation of thermal and environmental random noise. Consequently, the system can readily accommodate high-speed, low-speed, or high-SNR acquisition modes without hardware modification. A maximum sampling rate of 3 kHz is attainable under the condition that the system is configured with a single probe and one sample is taken per cycle.

In each time slot, one probe acts as the transmitter (TX) and activates its LEDs, while the remaining cascaded probes act as receivers (RX) for parallel synchronous multichannel acquisition. Each probe simultaneously performs signal acquisition and transmits the previously captured data frame to the hub, eliminating idle intervals and preserving a consistent high sampling rate over extended monitoring periods. Upon aggregating data from all probes, the hub compiles and packets the consolidated dataset and forwards it to the host computer via USB, supporting both offline and real-time signal analyses.

The modular cascaded hardware architecture allows optical detection channels to be formed between adjacent probes. The standardized layout guarantees a consistent SDS across the probe array and yields a spatially uniform detection network, as illustrated in Figure 4a. NIRSLINK supports up to eight cascaded probes and forms 52 valid detection channels with a fixed spacing of 30 mm. Channels with excessive SDS are excluded from analysis because severe optical attenuation reduces their reliability for physiological interpretation, as shown in Figure 4b [21,22].

#### 2.3.2. Adaptive Closed-Loop Parameter Tuning

Closed-loop control balances detection sensitivity against the risk of signal saturation. The system coordinates LED luminous intensity and signal amplification gain so that the acquired analog signals remain within the optimal ADC input range. Conventional manual tuning of gain and intensity is labor-intensive and time-consuming, and parameters optimized for one participant rarely transfer directly to another because tissue optical properties vary across individuals. Manual tuning is therefore poorly suited to adaptive and versatile wearable NIRS acquisition [23,24].

The tuning procedure consists of two sequential stages: coarse tuning and fine tuning. Algorithm 1 details the workflow. Tuning is performed while the probes are attached to the skin and the participant remains stationary, reducing motion-related bias. During coarse tuning, the LED drive current is fixed at 40 mA, and the system scans four available gain modes to select the configuration whose output voltage is closest to the predefined target range. After the gain is locked, fine tuning begins. A successive approximation algorithm adjusts the DAC output voltage to regulate LED drive current and optical power.
**Algorithm 1.** Two-Stage Tuning for Closed-Loop Control Set fixed LED driving current *I_LED_* = 40 mA. Set preset target output voltage *V*. Define four candidate gain combinations *A* = {*a_1_*,*a_2_*,*a_3_*,*a_4_*}. Coarse tuning stage:  **For** each *a* ∈ *A*:  Apply gain *a*, record output voltage *V*(*a*). Select optimal coarse gain:   a* = arg min_a_|*V(a)* − *V*| Lock fixed gain *a* = a*. Fine-tuning stage: Initialize *N*-bit DAC register *D* = 0, highest bit index *b* = *N* − 1. **While** *b* ≥ 0:  Tentatively set bit *b* of *D* to logic 1.  **If** *V(b)* < *V*:   Retain bit *b* = 1 in register *D*.  **Otherwise**:   Clear bit *b* to logic 0 in register *D*.  *b* ← *b* − 1 (switch to next lower bit). Output tuned gain a* and optimized DAC register *D*.

### 2.4. Shielding and Encapsulation

NIRSLINK employs separate rigid and flexible PCBs to enhance optical coupling and wearing stability. Figure 5a shows the cross-sectional structure of the designed probe. The front-end optical sensing module is assembled on an FPC and equipped with a spring-loaded floating mechanism that supports three-dimensional conformal contact with uneven skin surfaces. The back-end signal processing circuitry is integrated on a rigid PCB and enclosed in a 3D-printed protective housing. Rubber casings maintain low-pressure contact with the skin while limiting ambient light interference.

The LEDs and PDs are positioned at the four corners of each probe, forming four independent detection channels, as shown in Figure 5b. Each optoelectronic component has an individual optical lens to emit or receive light. Figure 5c shows the prototype. Its miniaturized and lightweight structure supports long-term wearable monitoring on multiple body regions.

### 2.5. Hardware Testing

SNR was adopted to quantify noise interference in optical signals. This parameter was calculated from raw light intensity measurements via the formula $20\;{log}_{10}(\bar{s}/\sigma_{s})$, where $\bar{s}$ is the mean signal magnitude and $\sigma_{s}$ refers to its standard deviation [13]. Tests on the phantom were conducted at a fixed SDS of 30 mm.

Dynamic range (DR) refers to the ratio between the maximum measurable optical power and the minimum detectable optical power of the system. Equipped with multi- level gain adjustment, the system can effectively avoid signal saturation and support signal acquisition under relatively high optical power.

### 2.6. Phantom Testing

Phantom experiments evaluated the dynamic detection capability and optical absorption response of NIRSLINK. Two controlled experiments were designed to emulate typical functional NIRS measurement scenarios: blood flow variations and hemoglobin concentration changes.

#### 2.6.1. Flow Phantom Test

Solid tissue-mimicking phantoms were fabricated from epoxy resin doped with titanium dioxide (TiO_2_) particles to reproduce the optical scattering properties of human soft tissue. During fabrication, a rubber tube with an outer diameter of 1 cm was embedded at different depths in the phantom to simulate heterogeneous vessel distributions [25,26]. Two NIRS probes were attached to the phantom surface, with their optical detection paths oriented perpendicular to the embedded tube, and both probes were operated at a sampling rate of 1 kHz.

The embedded tube was connected through flexible tubing to a peristaltic pump and a reservoir containing TiO_2_ aqueous solution, forming a closed-loop circulation system. Flow velocity inside the tube was controlled by adjusting the pump operating voltage in 2 V steps, thereby mimicking cerebral blood flow velocities under different physiological conditions.

**Figure 6:**
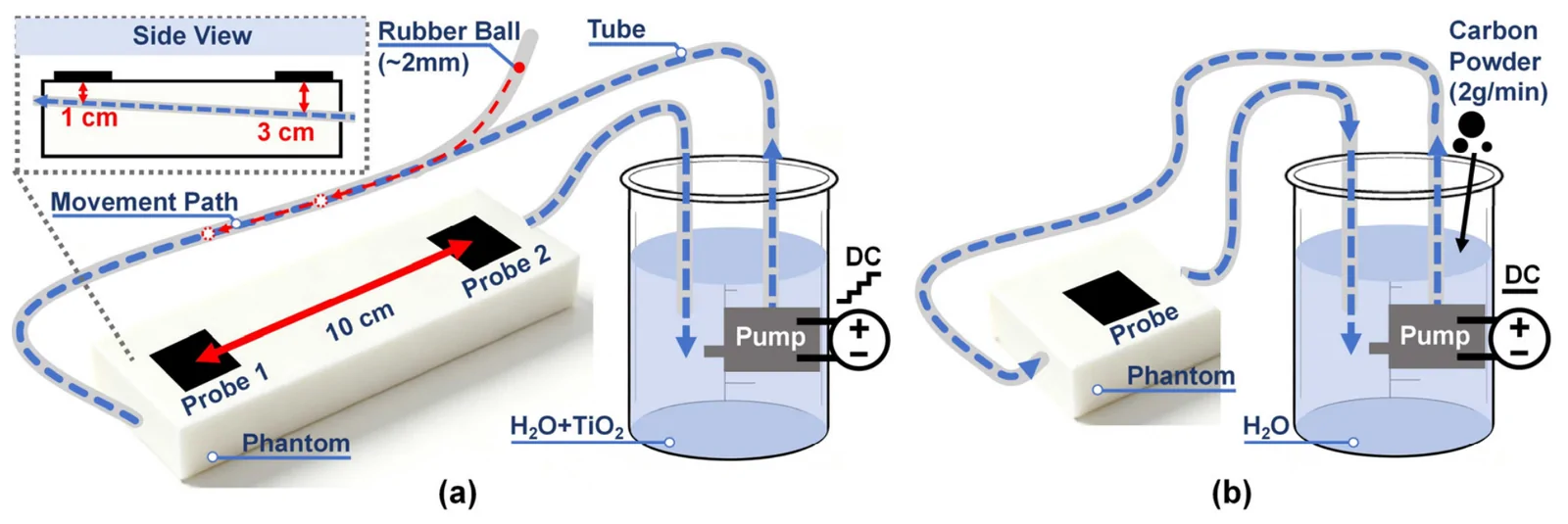
Experimental configurations for phantom validation: (**a**) Flow phantom setup: a closed-loop circulation system with a tissue-mimicking phantom and moving ball to evaluate temporal and spatial resolutions. (**b**) Absorption phantom setup: stepwise addition of carbon powder to the circulating medium to assess sensitivity to optical absorption changes.

To evaluate detection of dynamic absorbing targets, a black rubber ball with a diameter of 2 mm was introduced into the circulation loop. Real-time optical signals were recorded as the ball passed sequentially beneath two spatially separated probes. This configuration enabled quantitative assessment of temporal resolution and spatial localization for dynamic hemodynamic events, providing a basis for capturing transient responses in functional brain imaging. Figure 6a shows the complete experimental setup.

#### 2.6.2. Absorption Phantom Test

A second phantom experiment used the same scattering background to characterize the response to optical absorption changes independently. The peristaltic pump operated at a fixed voltage to maintain constant flow velocity, eliminating flow-induced signal fluctuations as a confounder. The circulating medium was initially water. Carbon powder was then added to the reservoir every 2 min at a fixed dose of 2 g, progressively increasing the absorption coefficient of the liquid to simulate hemoglobin concentration changes during neural activation. Optical signals from the NIRS probes were recorded continuously. Figure 6b shows the experimental configuration.

### 2.7. Human Subject Testing

In vivo human experiments were further conducted to assess the capability of NIRSLINK in detecting hemodynamic fluctuations and tissue oxygenation changes. Five physiological paradigms were employed, including the Valsalva maneuver, forearm vascular occlusion, a two-back working memory task, PWV measurement, and a motion feasibility test.

#### 2.7.1. Valsalva Test

All human experiments were performed in a quiet, dimly lit indoor environment. A total of three healthy adult participants (two males and one female) were recruited in this study, with a mean age of 28.3 ± 1.7 years. All participants were right-handed nonsmokers and had no history of chronic cardiovascular, cerebrovascular, metabolic, neurological, or cutaneous diseases, as well as no prior history of craniocerebral trauma, peripheral vascular lesions, or hypertension (all subsequent human experiments followed this enrollment criterion). Participants remained seated comfortably before testing to stabilize baseline physiological states. Three NIRS probes were attached to the scalp over the prefrontal cortex, with firm skin contact but without mechanical compression or probe displacement. Complete head fNIRS data during the Valsalva maneuver were acquired from the three participants at one-hour intervals, and three repeated measurement sessions were conducted in total.

**Figure 7:**
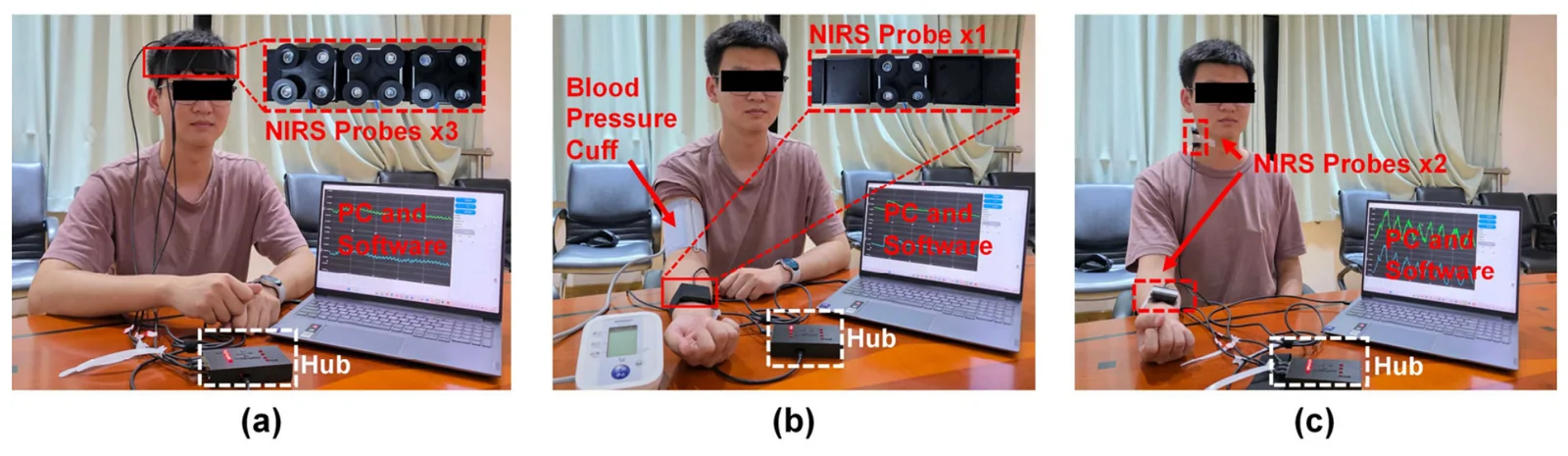
In vivo experimental paradigms: (**a**) Valsalva maneuver: three probes placed on the prefrontal scalp to record cerebral hemodynamic responses. (**b**) Vascular occlusion test: a probe attached to the forearm and a pressure cuff on the upper arm to induce transient ischemia and reperfusion. (**c**) PWV test: two NIRS probes positioned over the carotid and radial arteries, with the subject maintained at rest, to acquire pulse waveforms for estimation of PWV.

Each trial began with a 30 s resting period to establish stable baseline signals. Participants then performed the Valsalva maneuver following standard protocols. Briefly, they inhaled gently, closed the glottis, held their breath, and contracted the thoracic and abdominal muscles to elevate intrathoracic and intra-abdominal pressure for a predetermined duration. After the pressure maintenance phase, participants gradually relaxed the muscles, reopened the glottis, and resumed normal respiration until all hemodynamic signals returned to baseline [27,28]. To suppress noise and preserve waveform fidelity, the NIRS system was configured with an eight-point averaging scheme, yielding an effective sampling rate of 30 Hz. The experimental setups are shown in Figure 7a.

#### 2.7.2. Vascular Occlusion Test

During the vascular occlusion test, participants remained seated with the upper limb relaxed. A total of five healthy adult participants (three males and two females) were enrolled for this measurement, with a mean age of 26.6 ± 1.5 years. Three consecutive and uninterrupted cycles of arterial–venous occlusion were performed. One NIRS probe was attached to the forearm surface, and a medical inflatable cuff was wrapped around the proximal end of the same limb. The system was configured to operate at an effective sampling rate of 30 Hz.

A 60 s baseline recording was obtained under resting conditions. The cuff was then inflated alternately to 160 mmHg and 100 mmHg, with each pressure level sustained for a fixed period and separated by recovery intervals. After each occlusion period, the cuff was gradually deflated to monitor hemodynamic reperfusion [29,30,31]. The experimental setups are shown in Figure 7b.

#### 2.7.3. PWV Test

For the PWV measurement, data were acquired from five healthy adult participants (four males and one female) with no cardiovascular or systemic diseases, with a mean age of 28.4 ± 1.1 years. Resting blood pressure prior to data collection was recorded for all participants, and all recordings were performed under sustained resting conditions. Dual-modal signals were synchronously collected: two NIRS probes, placed over the carotid and radial arteries for PWV estimation, operated with a 10-point averaging mode yielding an effective sampling rate of 166 Hz (probe arrangement illustrated in Figure 7c). Many recent cardiovascular monitoring studies have verified the feasibility and effectiveness of utilizing synchronized ballistocardiogram (BCG) and photoplethysmography (PPG) signals for pulse timing evaluation. The J fiducial point extracted from chest BCG precisely corresponds to the instant of aortic valve opening and blood ejection, avoiding pre-ejection period bias contained in electrocardiogram (ECG)-derived pulse arrival time (PAT) values, and it has been validated to align well with impedance cardiogram (ICG) gold-standard cardiac markers in multiple controlled experiments [32,33,34]. Simultaneously, a chest-attached accelerometer recorded the z-axis BCG, and a fingertip PPG sensor captured the peripheral pulse waveform in parallel. Both devices were sampled at 1 kHz, with all waveforms strictly time-synchronized. The peak-to-peak interval between the synchronized BCG and PPG signals, denoted as PTT_BP_, was used for cross-modal comparison.

#### 2.7.4. Two-Back Task

**Figure 8:**
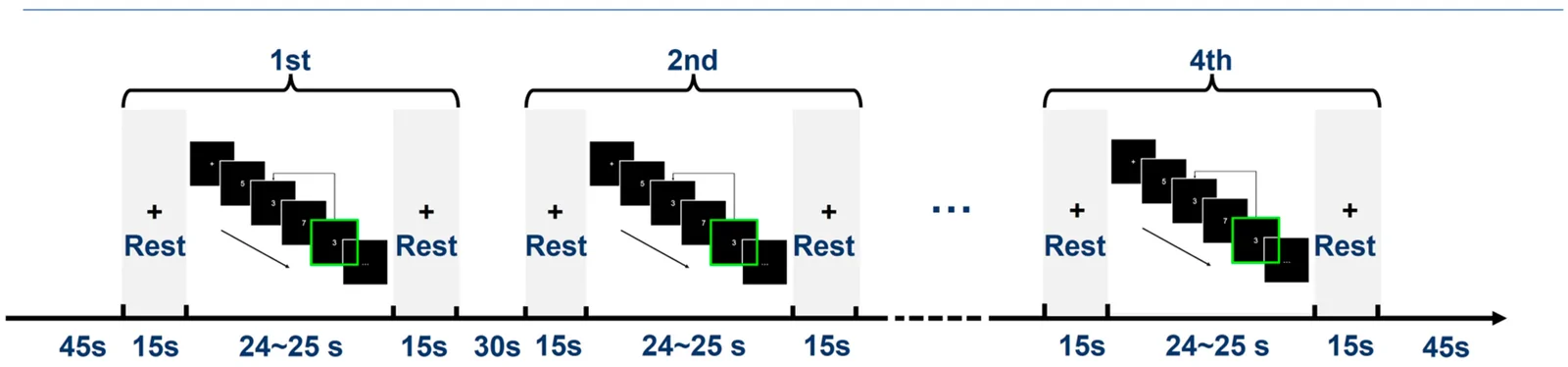
Timeline of the two-back working memory task. The protocol consists of a 45 s baseline and four consecutive two-back blocks separated by 30 s recovery intervals.

A total of five healthy adult participants (three males and two females) were recruited, with a mean age of 26.6 ± 1.7 years. The optical probe configuration was identical to that used in the Valsalva maneuver test (Section 2.7.1). Each experimental session followed a fixed protocol comprising a 45 s baseline recording, followed by four consecutive two-back working memory blocks with identical task parameters. Adjacent blocks were separated by a 30 s recovery interval to allow the haemodynamic signals to return towards pre-stimulus levels. The experimental setup and temporal sequence are illustrated in Figure 8.

#### 2.7.5. Motion Feasibility Test

A preliminary motion feasibility experiment was conducted using five wearable NIRS probes. Three probes were secured on the forehead, while the other two were attached to the left and right wrists, respectively. All probes remained in position throughout the experiment. Each experimental session followed a fixed 240 s protocol consisting of a 60 s initial resting period, 60 s of repeated head turning, 60 s of stepping in place, and a final 60 s post-motion resting period. Signal quality was evaluated separately for each probe, wavelength, and experimental stage using the coefficient of variation (CV): $CV\;=\sigma_{I}/\bar{I}\times 100\%$, where $\bar{I}$ and $\sigma_{I}$ denote the mean and standard deviation of the raw optical intensity within the corresponding 60 s stage. Channels with a valid data proportion of at least 95%, positive mean optical intensity, and a CV not exceeding 15% were considered usable [35].

## 3. Results

### 3.1. Hardware Characterization and Phantom Measurements

Table 1 summarizes the hardware parameters and performance metrics of NIRSLINK.

**Figure 9:**
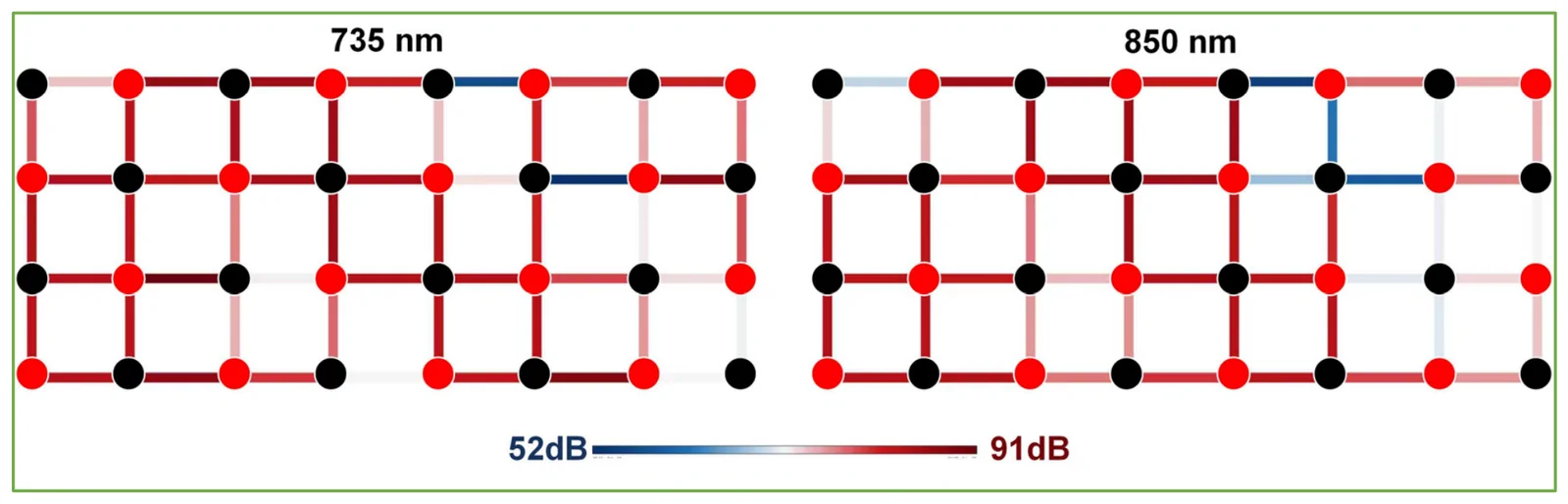
Spatial distribution of the SNR across the network comprising eight NIRS probes and 52 dual-wavelength measurement channels during phantom testing. The (**left**) and (**right**) panels show the channel-wise SNR distributions at 735 and 850 nm, respectively. Colors ranging from dark blue to dark red indicate an increasing SNR. The comparable SNR distributions at the two wavelengths demonstrate that the full eight-probe network maintains consistently high signal quality across its spatially distributed channels.

As shown in Figure 9, the signal quality of a network comprising eight NIRS probes and 52 dual-wavelength measurement channels was evaluated using a phantom. The figure presents the spatial distributions of the channel-wise SNR at 735 and 850 nm, with colors ranging from dark blue to dark red, indicating an increasing SNR. The mean SNRs were 79.38 ± 6.94 dB at 735 nm and 77.84 ± 7.05 dB at 850 nm, with an overall mean of 78.61 ± 7.03 dB across both wavelengths. The relatively consistent SNR distribution indicates that the multi-probe network provides reliable signal acquisition in phantom measurements, supporting its use in subsequent experiments and signal analyses.

Although the NIRSLINK supports a maximum sampling rate of 3 kHz, the effective sampling rate decreases as the number of cascaded probes increases. Table 2 summarizes the quantitative relationship between the cascade count and the actual sampling rate. This performance degradation is primarily attributable to the escalating data throughput burden on the entire transmission link. With more probes in cascade, the volume of data transmitted from each probe to the hub via the SPI bus, and subsequently from the hub to the host computer, grows proportionally. This increased data flow not only prolongs the transmission time but also consumes excessive bus communication timing resources, thereby constraining and reducing the effective sampling speed of the multi-probe cascaded system.

**Figure 10:**
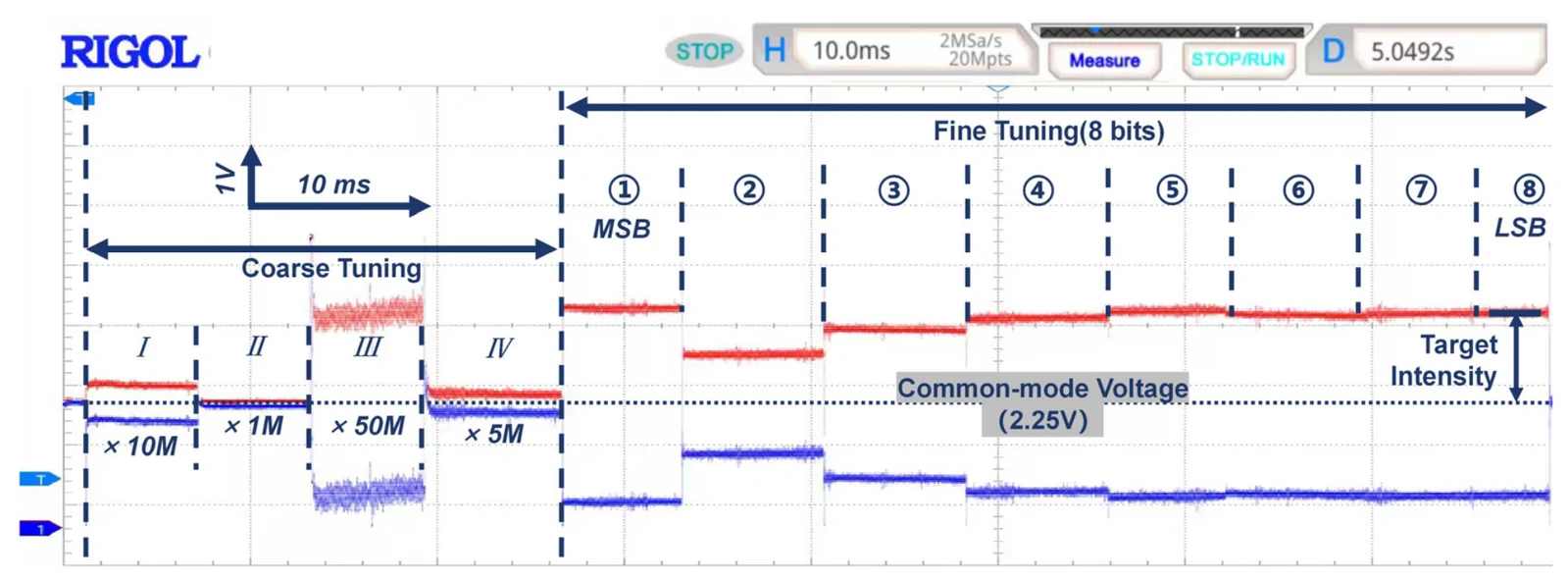
Measured differential output waveforms during closed-loop tuning, illustrating the two-stage tuning process. The red and blue traces converge symmetrically toward the target voltage. Both traces converge within 90 ms without manual adjustment.

Figure 10 presents the differential output waveforms collected from a probe mounted on a phantom during closed-loop tuning, which validates the efficacy of the two-stage tuning approach. During coarse tuning (Regions I–IV), the system sequentially switches among four preset gain settings (×10 M, ×1 M, ×50 M, and ×5 M) to identify the configuration with the minimum residual error relative to the target voltage. After coarse gain selection, an 8-bit binary-weighted fine-tuning sequence (Regions 1–8) proceeds from the most significant bit (MSB) to the least significant bit (LSB) to cancel the residual offset. After these four coarse-tuning steps and the subsequent eight fine-tuning steps, the system converges to the target voltage within 90 ms. The differential output voltage thus approaches the target value in a stepwise manner, experimentally validating the closed-loop tuning.

To further corroborate the robustness of the proposed closed-loop tuning strategy, we conducted systematic validation experiments with the probe mounted on various regions of the human body, and these measurements were performed on three different participants. Two comparative test groups were established. In Group I, output voltages were sampled after adaptive gain tuning with a target voltage of 2.5 V. Measurements revealed that under automatic tuning, upon completion of a single full tuning sequence at all test sites, the outputs converged to around 2.5 V within 90 ms. In Group II, the optical driving current was fixed at 80 mA, and a fixed gain of 5 MΩ was adopted for voltage output acquisition. To mitigate the impact of inherent fluctuations in physiological blood flow signals, all data points presented in the associated table were averaged over a 1 s acquisition duration. The comprehensive experimental outcomes are summarized in Table 3. Group I with adaptive tuning maintains output voltages close to the 2.5 V target across all measured anatomical sites. In contrast, Group II without adaptive tuning exhibits pronounced voltage deviations among different body regions. Notably, under fixed optical drive current and gain, the head yields the lowest output voltage, whereas the thigh produces the highest. This discrepancy stems from tissue-specific optical properties: signals measured at the head traverse the skull, which introduces significant attenuation and reduces the reflected light captured by the probe. Conversely, the thigh features thicker soft tissue and fewer highly absorptive bony structures, resulting in substantially stronger reflected signals. Even with closed-loop adjustments, minor residual deviations from the 2.5 V target persist across sites, partly attributable to site-dependent physiological blood flow fluctuations. Overall, these findings demonstrate that the proposed closed-loop tuning strategy effectively suppresses amplitude variations induced by heterogeneous tissue optics, maintaining outputs within a narrow voltage range across diverse human tissues, which is critical for mitigating signal saturation and low-amplitude noise in multi-site fNIRS acquisitions.

**Figure 11:**
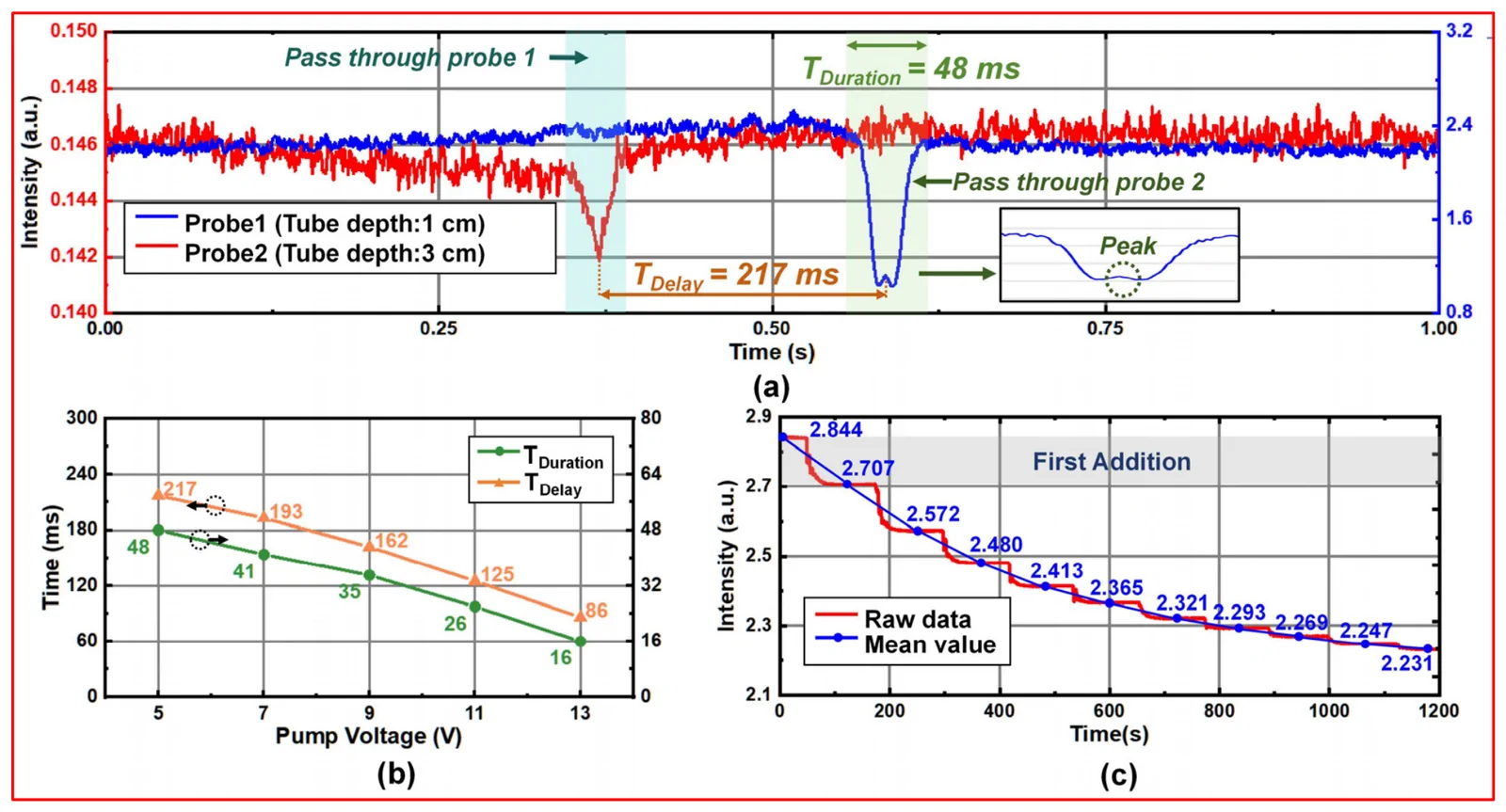
Phantom test results: (**a**) Temporal intensity traces from two probes in the flow phantom, showing sharp dips as a black ball passes beneath probe 1 and probe 2. (**b**) Transit time (*T_Delay_*) and interaction duration (*T_Duration_*) versus pump voltage, exhibiting an approximately linear decrease. (**c**) Signal attenuation upon successive additions of carbon powder. The resolved transit events and monotonic absorption-dependent attenuation demonstrate sensitivity to both millisecond-scale dynamic perturbations and progressive changes in optical absorption.

To evaluate the temporal resolution and detection sensitivity of the NIRSLINK system under dynamic scattering perturbations, we employed a flow phantom with a moving absorbing ball as a localized test object. Time-domain intensity traces from two probes are displayed in Figure 11a. Both traces exhibit sharp dips as the black rubber ball passes sequentially beneath probe 1 and probe 2, with the magnified insets revealing the complete transient attenuation profiles induced by the ball. Owing to uneven tube embedding, probe 1 possesses a shallower detection depth, resulting in weaker attenuation of scattered light and consequently a higher baseline intensity compared with probe 2.

Figure 11b illustrates the variations of *T_Delay_* and *T_Duration_* as the drive voltage is incremented from 5 V to 13 V in 2 V steps. *T_Delay_* denotes the time difference between the ball passing beneath the two probes, while *T_Duration_* represents the interaction duration between the ball and probe 1. Both parameters decrease in an approximately linear fashion over this range, with *T_Delay_* dropping from 217 ms to 86 ms. Notably, even at the highest voltage (13 V), distinct dips remain clearly resolvable, confirming that NIRSLINK captures full transient responses without loss of temporal fidelity, even at elevated ball velocities.

Beyond localized scattering perturbations, we further examined the system’s responsiveness to progressive changes in background optical absorption by sequentially introducing carbon powder into the circulating medium. The resulting signal evolution, presented in Figure 11c, exhibits a monotonic decrease in intensity over the entire addition sequence. Of particular note, the first addition induces a pronounced drop, which can be ascribed to the inverse relationship between solution concentration and detected intensity.

### 3.2. Human Subject Measurements

**Figure 12:**
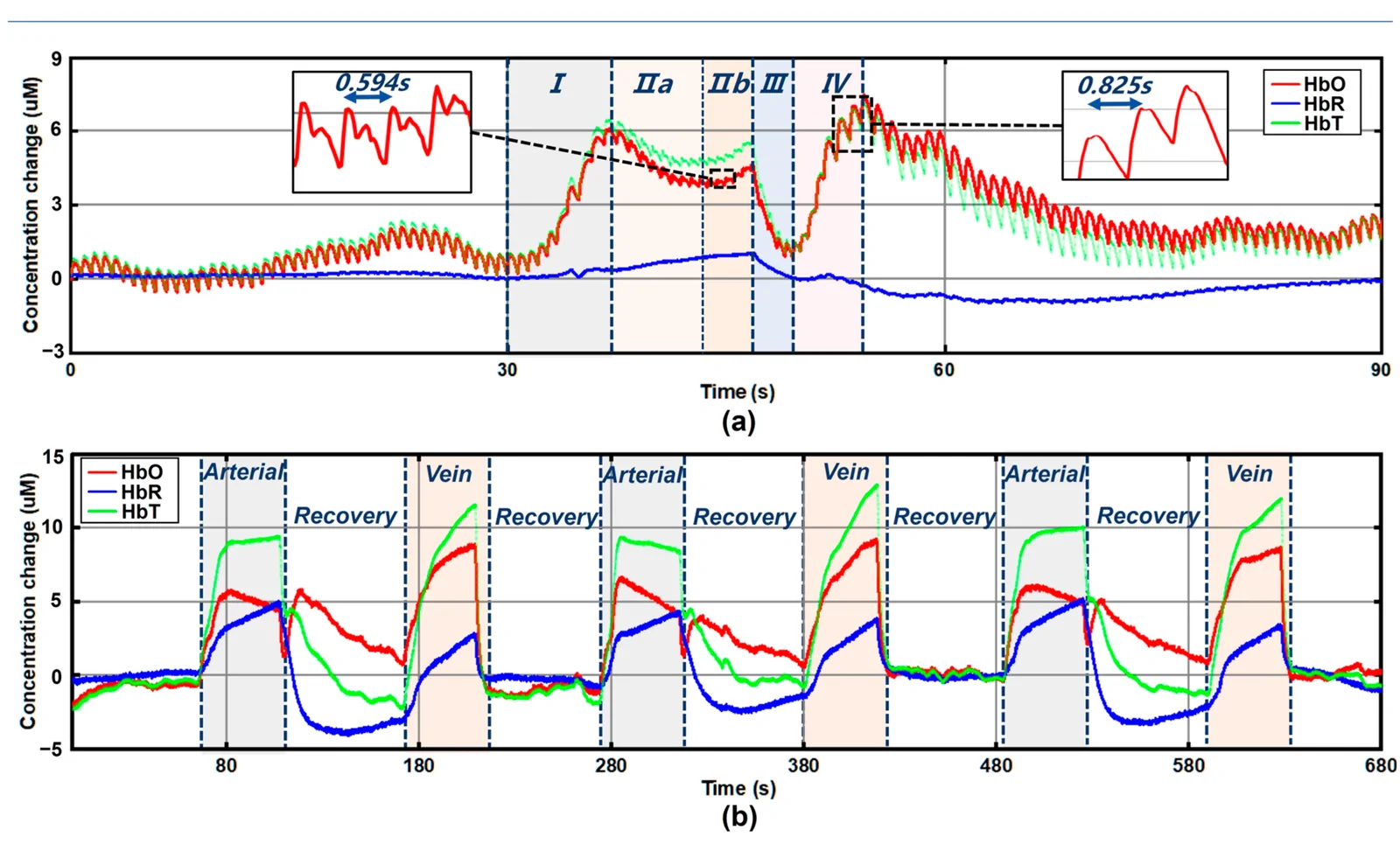
In vivo hemodynamic responses: (**a**) Prefrontal concentration changes in HbO, HbR, and HbT during the Valsalva maneuver, showing phase-dependent variations and superimposed cardiac oscillations. (**b**) Forearm responses during occlusion. The phase-specific cerebral response and the distinct arterial-versus-venous occlusion patterns show that NIRSLINK can distinguish physiologically different hemodynamic processes at cerebral and peripheral sites.

The Valsalva maneuver, a standardized respiratory challenge that acutely elevates intrathoracic and intracranial pressures, is widely employed to probe cerebral hemodynamic regulation and autonomic control. The prefrontal hemodynamic responses elicited during this maneuver are depicted in Figure 12a, which tracks concentration changes in oxygenated (HbO), deoxygenated (HbR), and total hemoglobin (HbT). Over this period, HbO demonstrated pronounced phase-dependent dynamics, with cardiac-related oscillations superimposed on the recorded waveform. These distinct phase-dependent hemodynamic trajectories correspond to the four classic physiological stages of the Valsalva maneuver: Phase I features transient aortic compression that elevates arterial blood pressure and HbO concentration; Phase IIa exhibits reduced venous return that lowers blood pressure alongside an HbO decline; Phase IIb presents an activated sympathetic response raising peripheral resistance and resulting in secondary HbO elevation; Phase III involves sudden intrathoracic pressure release that induces a simultaneous drop in blood pressure and HbO; Phase IV shows rebound venous return, triggering transient HbO overshoot prior to recovery to baseline.

Given the distinct stage-wise characteristics observed in the waveforms, we selected the HbO concentration changes at different stages for further analysis. The corresponding mean values and standard deviations are summarized in Table 4. Although the absolute signal amplitudes varied across trials and participants, the overall rising-and-falling trends at each stage were consistent, and this temporal response pattern could be reproduced across repeated trials within each subject. To evaluate the test–retest reliability of the stage-specific signal amplitudes, we further calculated the single-measure intraclass correlation coefficient [*ICC*(3,1)]. The results showed that stage IIb achieved excellent measurement reliability (*ICC* = 0.851, *p* < 0.01), whereas no statistical significance was observed for the remaining stages. This can be largely attributed to the pronounced confounding effects of the Valsalva maneuver, including variable breath-holding effort and inter-individual physiological differences, which tend to modulate absolute amplitude rather than waveform shape. Although the overall trends remained consistent, the considerable dispersion in absolute HbO amplitudes limited the repeatability of single-point amplitude measurements for most stages.

In contrast, the forearm hemodynamic responses under occlusion protocols, shown in Figure 12b, reveal distinct patterns: arterial occlusion induces a progressive decline in HbO alongside a concurrent rise in HbR, whereas venous occlusion drives simultaneous increases in both HbO and HbR. The slopes of HbO and HbR during the rising and falling phases under arterial and venous occlusion were analyzed in this study. The measured results from five participants were as follows: HbR slope under arterial occlusion: 0.0527 ± 0.0077 μM/s; HbR slope under venous occlusion: 0.1924 ± 0.0176 μM/s; HbO slope under arterial occlusion: −0.0426 ± 0.0471 μM/s; HbO slope under venous occlusion: 0.6490 ± 0.1826 μM/s. Statistical comparisons using paired-sample *t*-tests were performed to evaluate slope differences between arterial and venous occlusion conditions. Although venous occlusion yielded numerically larger HbR and HbO slopes, no statistically significant differences were observed between the two occlusion modes (*p* > 0.05). This absence of statistical significance could be attributed to the limited sample size, inter-subject variability in forearm tissue composition, and trial-to-trial subtle variations in cuff pressure and occlusion completeness. Larger HbR and HbO slopes were obtained under venous occlusion, which reflects distinct modulatory effects of the two occlusion modes on forearm tissue oxygenation signals.

**Figure 13:**
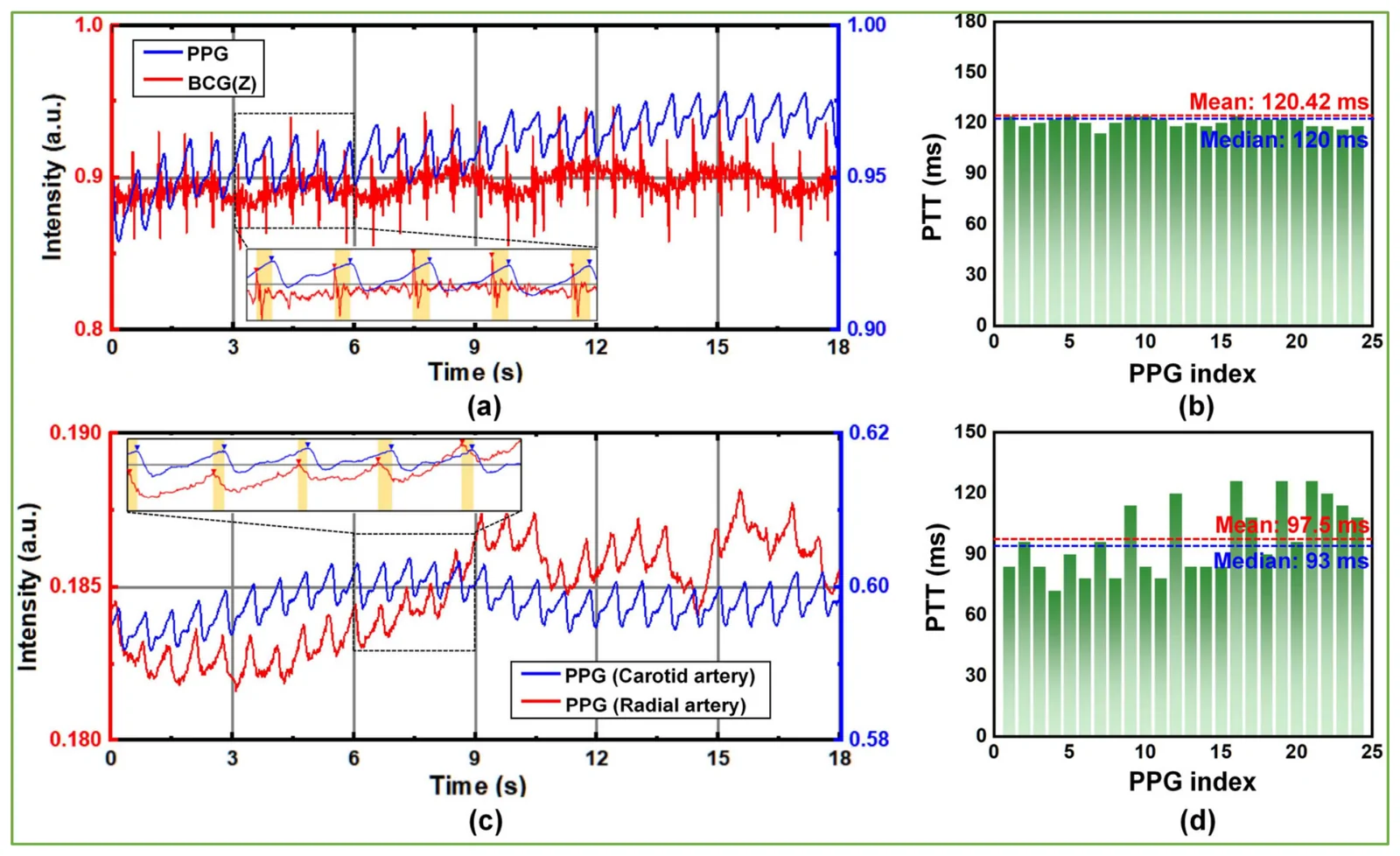
Synchronized PTT measurements obtained using the reference BCG-PPG configuration and NIRSLINK: (**a**) Simultaneously recorded chest BCG (z-axis acceleration) and fingertip PPG signals over 18 s; the inset shows the peak-to-peak timing interval between corresponding cardiac cycles. (**b**) Beat-to-beat PTT_BP_ values obtained from the reference measurement (120.42 ± 2.71 ms; median: 120 ms). (**c**) Carotid and radial pulse waveforms simultaneously recorded by two NIRS probes; the inset illustrates the inter-site pulse delay used to determine PTT. (**d**) Beat-to-beat carotid-radial PTT measured by NIRSLINK (97.50 ± 17.34 ms; median: 93 ms).

Figure 13 presents the PTT results obtained from one representative participant using NIRSLINK together with a synchronized reference measurement. As shown in Figure 13a, the chest BCG and fingertip signals exhibited stable periodic cardiac features during the 18 s resting-state recording. The resulting PTT_BP_ was 120.42 ± 2.71 ms, corresponding to a within-recording coefficient of variation of 2.25% (Figure 13b). Meanwhile, two NIRS probes positioned over the carotid and radial arteries simultaneously recorded periodic arterial pulse waveforms with a discernible distal delay (Figure 13c). The carotid–radial PTT was 97.50 ± 17.34 ms, with a coefficient of variation of 17.78% (Figure 13d). Using the measured carotid-to-radial path length of 0.80 m, the mean PTT yielded an estimated segmental PWV of approximately 8.21 m/s. Likewise, adopting the measured heart-to-fingertip path length of 0.96 m and the averaged PTT_BP_, the estimated PWV_BP_ was 7.97 m/s. Although the two PWV estimates are comparable, considerable discrepancies still exist between them. Both the reference measurement and NIRSLINK identified approximately 24 cardiac cycles within the 18 s recording, corresponding to an average heart rate of approximately 80 beats/min. The consistent cardiac-cycle detection supports the capability of NIRSLINK to capture arterial pulsations and resolve inter-site pulse delays.

**Figure 14:**
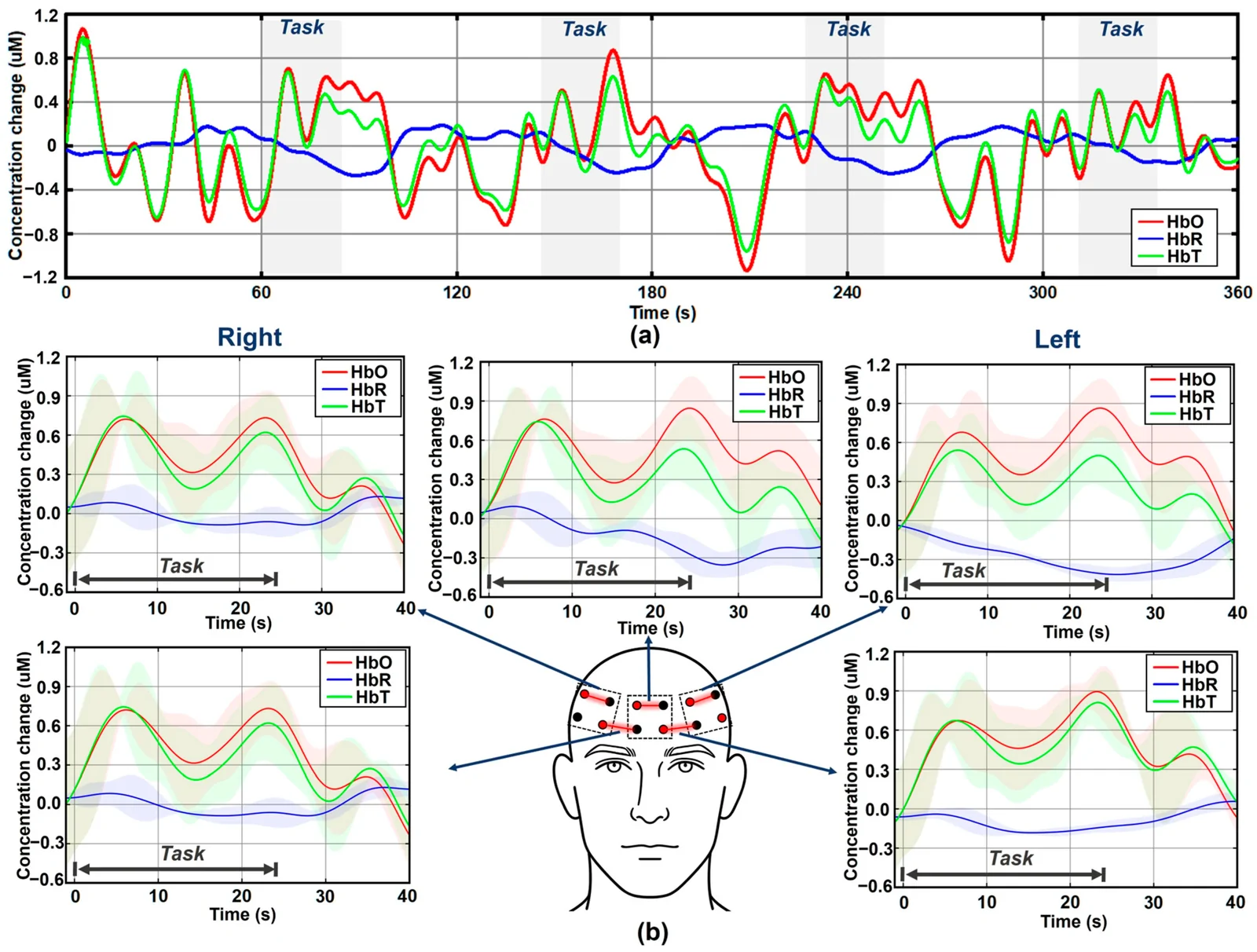
HRF during the two-back cognitive task: (**a**) Continuous traces of HbO and HbR from a representative channel over the full session, including baseline, four task blocks, and recovery periods. (**b**) Averaged task-related HRF for five selected channels, including both intra- and inter-probe channels. After systemic signal correction, the observed increases in oxygenated hemoglobin and accompanying decreases in deoxygenated hemoglobin across task blocks and channels confirm that the system can detect spatially distributed prefrontal activation.

The two-back working-memory task is a well-established paradigm for probing executive function and prefrontal cortical engagement, with task-evoked haemodynamic changes providing an indirect measure of regional neural activity. We adopted the functional systemic signal separation algorithm [36] to decompose mixed fNIRS signals. All data from five participants were processed with this signal decomposition approach to reduce superficial physiological artifacts. Figure 14a shows the corrected continuous changes in oxygenated HbO and deoxygenated HbR concentrations recorded from a representative channel; after superficial artifact removal, HbO rises during task blocks, HbR declines consistently throughout all task periods and gradually rebounds after task offset, which matches canonical cortical activation patterns. Figure 14b illustrates the optode configuration and delineates three prefrontal regions of interest: the left dorsolateral prefrontal cortex (DLPFC), medial prefrontal cortex (mPFC), and right DLPFC. The averaged corrected haemodynamic response functions (HRFs) of five selected channels, comprising both intra-probe and cascaded inter-probe configurations, are also presented. Solid lines denote group-mean concentration changes, and shaded bands represent a ±1 standard deviation across participants.

Across the selected channels, the task-evoked response was characterized by an increase in HbO accompanied by a reduction in HbR, consistent with the canonical haemodynamic signature of prefrontal cortical recruitment. The HbO time courses exhibited a reproducible multiphasic profile: an initial increase occurred at approximately 6–7 s, followed by a second, generally larger peak at approximately 23~24 s, coinciding with the end of the two-back task. A smaller third fluctuation was observed in several channels at approximately 34–35 s. Among the channels examined, CH10 (as shown in the upper-right subplot of Figure 14b) exhibited the largest HbO response, reaching 0.845 ± 0.15 uM at approximately 23.2 s.

In the motion experiment, no data dropout was observed across all channels during the entire acquisition procedure, and all five probes maintained continuous dual-wavelength optical-signal acquisition throughout the head-turning and stepping stages. During initial rest, the mean CV ranged from 0.68% to 1.61% at 735 nm and from 0.76% to 2.85% at 850 nm. During head turning, the corresponding ranges were 0.84–2.13% and 0.77–3.61%, respectively. A larger increase was observed during stepping, for which the mean CV ranged from 3.14 to 6.18% at 735 nm and from 4.42 to 7.27% at 850 nm. Nevertheless, all mean CV values remained below the predefined 15% threshold. During post-motion recovery, the CV decreased to 1.88–2.76% at 735 nm and 2.30–3.52% at 850 nm.

**Figure 15:**
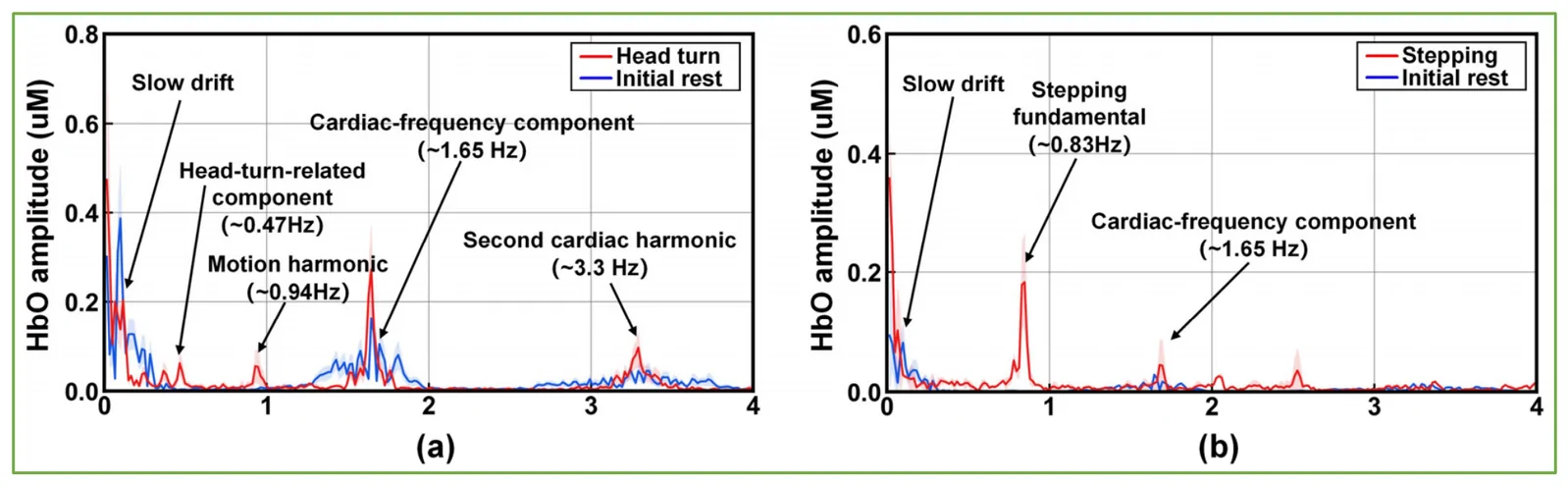
Frequency-domain comparison of the unfiltered HbO signals under resting and motion conditions. (**a**) Spectra of the three forehead-mounted probes during initial rest and repetitive head turning. (**b**) Spectra of the two wrist-mounted probes during initial rest and stepping in place. The blue and red solid lines represent the mean spectra across probes during the initial resting and motion stages, respectively, and the shaded regions indicate ±1 standard deviation. After removal of the stage-specific mean and application of a Hann window, the amplitude spectra were calculated using a one-sided fast Fourier transform.

As shown in Figure 15, the frequency-domain results revealed task- and location-dependent motion-associated components. For the forehead-mounted probes, head turning produced an enhanced component near 0.47 Hz and a motion-related harmonic near 0.94 Hz. Components near 1.65 and 3.30 Hz were also observed and correspond to the cardiac frequency component and its second harmonic, respectively. For the wrist-mounted probes, stepping produced a prominent component near 0.83 Hz; this component was substantially enhanced compared with the initial resting condition.

Overall, stepping produced a greater increase in signal variability than head turning, particularly for the wrist-mounted probes. However, continuous signal acquisition was maintained for all five probes, all mean CV values remained below 15%, and signal variability decreased following cessation of movement. These results provide preliminary support for the feasibility of the proposed system under moderate body motion conditions.

## 4. Discussion

Existing wearable NIRS systems have adopted modular mechanical frameworks, distributed front-end readout circuits, and scalable optode arrays; however, each of these designs has been optimized for only a single performance aspect, and achieving simultaneous integration of cross anatomical optical conformability, high-speed multichannel synchronous acquisition, and fully autonomous adaptive signal tuning on one hardware platform remains difficult, a challenge that precisely motivates the multi-objective co-optimization principle guiding the design of the proposed NIRSLINK system. Most current modular probes are custom-built for cranial measurements, with configurations tailored to scalp geometry, and published validations demonstrating concurrent hemodynamic recordings across multiple body sites are scarce; in contrast, the flexible probes of NIRSLINK, equipped with floating optical assemblies, support synchronous signal acquisition over curved surfaces, including both the scalp and the extremities. For multichannel recordings, conventional distributed architectures suffer from pronounced sampling performance degradation when multiple probes operate in parallel, which limits the ability to capture fast transient physiological events, whereas in the present system, signal digitization is performed locally at each probe; although the effective sampling rate decreases as more probes are cascaded, the intrinsically robust acquisition capability of each individual probe still ensures acceptable sampling speeds under multi-probe synchronous operation. Regarding signal tuning, mainstream devices rely on manual adjustment of amplification and light source parameters for different subjects and measurement sites, and simple single-stage auto-regulation cannot adequately suppress signal fluctuations caused by inter-subject and inter-site variations in tissue optical properties; to address this, the present work proposes a two-stage closed-loop tuning strategy that coordinately regulates optical and electrical parameters, thereby confining raw detected signals within the optimal acquisition range without requiring operator intervention. In summary, existing modular wearable NIRS platforms predominantly emphasize hardware scalability, whereas very few designs have achieved the concurrent optimization of multi-site conformability, high-speed synchronous sampling, and adaptive tuning. The detailed performance indicators of the proposed NIRSLINK system and a comprehensive quantitative comparison with wearable NIRS systems reported in recent years are presented in Table 5.

Flow phantom tests further demonstrate the ability of NIRSLINK to resolve millisecond-scale transient events and subtle optical differences caused by depth heterogeneity. Waveforms recorded by probes over shallow embedded tubing show distinct positive peaks, which can be explained by the canonical banana-shaped photon propagation path in NIRS [43,44]. Photon flux is distributed unevenly along curved trajectories, so the attenuation contribution of an absorber depends on the local photon flux weight of the path segment it crosses. Higher photon flux density in superficial tissue amplifies optical perturbations from absorbers and produces prominent positive peaks in the recorded waveforms. These results confirm fine resolving power for photon tissue interactions at high temporal resolution.

Standardized in vivo physiological measurements provide validation that NIRSLINK captures hemodynamic signals. During the Valsalva maneuver, prefrontal HbO concentrations show phase-wise variations that closely follow established cardiovascular physiology. In Phase I, elevated intrathoracic pressure compresses the aorta, increasing arterial blood pressure and HbO. In Phase IIa, impaired venous return reduces atrial filling, accompanied by decreases in blood pressure and HbO. Further hypotension activates arterial baroreceptors, increases sympathetic outflow, elevates heart rate and peripheral vascular resistance, and induces secondary increases in blood pressure and HbO (Phase IIb). In Phase III, termination of breath holding abruptly reduces intrathoracic pressure, and blood pressure and HbO decrease simultaneously [45,46,47]. In Phase IV, restored venous return triggers transient sympathetic activation, producing an HbO elevation followed by a vagally mediated recovery to baseline.

Forearm vascular occlusion tests show that recovery after arterial occlusion is longer than recovery after venous occlusion. Arterial cuff compression interrupts tissue blood supply, and continued cellular oxygen consumption causes severe tissue hypoxia and HbR accumulation. Extended reperfusion is therefore required to clear accumulated HbR and restore baseline oxygenation. In contrast, venous occlusion blocks venous outflow while arterial inflow continues, producing only mild regional blood stasis [48,49,50]. After cuff release, pooled venous blood drains rapidly back into systemic circulation, enabling fast recovery of the hemodynamic signals. These divergent response patterns validate the ability of NIRSLINK to discriminate tissue oxygenation states and support its use in peripheral microcirculation assessment.

The dual-site pulse waveforms recorded by NIRSLINK demonstrate that the proposed system can resolve inter-site pulse arrival delays on a millisecond timescale. Many conventional NIRS systems operate at sampling rates of approximately 10 Hz, limiting their ability to capture rapid systolic features and quantify PTT on a beat-to-beat basis. Simultaneous recording of the carotid and radial pulse waveforms within a common acquisition architecture enables synchronized monitoring across multiple vascular sites and reduces timing uncertainty associated with independently operated devices. The synchronized BCG-PPG measurement provided an independent reference for cardiac-cycle detection, and the agreement in the number of detected cardiac cycles and derived heart rate supports the temporal fidelity of the system. Furthermore, combining inter-site PTT with a defined propagation distance permits estimation of segmental PWV, providing a potential means of characterizing vascular properties along a specific arterial path. Collectively, these findings support the feasibility of the proposed platform for synchronized multi-site pulse-wave acquisition and vascular timing assessment. The greater beat-to-beat variability of the NIRS-derived PTT likely reflects a combination of anatomical and measurement-related factors. Fingertip green-light PPG is sensitive to pulsatile blood volume changes in a well-perfused superficial vascular bed and generally provides prominent pulse features. In contrast, the NIRS probes collect diffusely reflected light from a larger tissue volume, with the arterial pulsation superimposed on a substantial non-pulsatile optical background. Pulse-feature identification at the carotid and radial sites may also be influenced by probe positioning, contact pressure, local waveform morphology, vascular wave reflection, and respiratory or neck motion. These factors may collectively contribute to the observed variability, and their individual contributions cannot be disentangled in the present study. In this experiment, five participants were enrolled to explore the inherent correlation between the extracted PWV and physiological hemodynamic characteristics reflected by wearable NIRS signals. Pearson correlation analysis was adopted to evaluate their association, yielding *r* = 0.31, *p* = 0.61, which indicated no statistically significant correlation. This outcome may be attributed to the limited sample size, inter-individual differences in baseline vascular properties, and the narrow dynamic range of vascular hemodynamic variations under resting conditions. In addition, measurement noise and estimation errors introduced by surface-based path-length approximation could further weaken the observed correlation. Future work will expand the participant cohort, adopt targeted physiological intervention protocols to enrich the variation range of vascular hemodynamic parameters, and optimize pulse-fiducial-point extraction as well as propagation-distance calibration to further explore the intrinsic linkage between PWV and vascular hemodynamic responses.

During the two-back cognitive task, task-evoked hemodynamic activation over the left DLPFC exceeded that over the right DLPFC (*t*(4) = 2.94, *p* = 0.043). This spatial asymmetry is consistent with functional specialization across prefrontal subregions: the left DLPFC is involved in short-term storage and maintenance of sequential verbal digit information, whereas the right DLPFC contributes to executive processes such as stimulus matching and inhibitory control [51,52,53].

The evaluation under motion conditions extended the system assessment from a static laboratory environment to body movement scenarios that are more representative of practical wearable applications. As expected, body movement increased the variability of the optical signals, with stepping producing a greater effect than repetitive head turning. This effect was particularly evident for the wrist-mounted probes, possibly because of arm swing, wrist rotation, local tissue deformation, and changes in probe–skin coupling. Despite the increased signal variability during movement, continuous acquisition was maintained across all five probes, and all mean CV values remained below 15%. Furthermore, the reduction in the CV during the post-motion resting stage suggests that the observed changes were predominantly associated with temporary movement rather than persistent communication failure or irreversible probe displacement. These findings provide preliminary support for the feasibility of continuous wearable measurements under moderate body movement conditions. Future studies will incorporate synchronized inertial measurements, larger participant cohorts, and further evaluations involving continuous walking and activities of daily living.

Several limitations remain. First, NIRSLINK does not include short SDS channels, which are important for removing extracerebral scalp hemodynamic artifacts through regression-based correction. The current system mitigates superficial interference mainly through spatial-consistency verification and signal-quality screening; measurement errors may therefore increase during long recordings or in participants with high cutaneous perfusion. Second, the current prototype does not include wireless communication modules such as Bluetooth. Wired cabling between probes and the main controller reduces wearability and limits body movement, making the system less suitable for dynamic monitoring scenarios that require unrestricted locomotion.

## 5. Conclusions

This study presents NIRSLINK, a modular cascaded wearable NIRS system that combines high-speed sampling with distributed front-end processing. Lightweight flexible probes, independent TIA, and two-stage closed-loop tuning enable multi-site deployment, low-noise acquisition, and wide, dynamic range adaptation. Phantom tests confirm high temporal resolution and reliable sensitivity to absorption changes, while the in vivo experiments validate the ability of the system to capture typical cerebral and peripheral hemodynamic responses. The current prototype is limited by the absence of short source detector channels and wireless connectivity. Future work will integrate short channel correction, multi-wavelength modules, and wireless communication to improve quantitative accuracy and mobility. Overall, NIRSLINK provides a robust platform for multi-site wearable hemodynamic monitoring in neuroscience and ambulatory healthcare applications.

## Figures and Tables

**Table 1:** Hardware specifications and performance of NIRSLINK.

**Physical Properties**	
Light source	2 LEDs (735 nm, 850 nm)
Light detector	2 PDs
SDS	30 mm
Size of NIRS probe	45 × 45 mm^2^
Weight of NIRS probe	31 g
Weight of hub	102 g
**Performance**	
Maximum probes	8
Maximum number of valid channels	52
Maximum sampling rate	3 kHz
**Sensor**	
End-to-end linearity (R^2^)	0.92 ± 0.07
Long-term stability (4 h)	−0.413 ± 0.13%/h
Thermal drift (25–40 °C)	−0.122 ± 0.08%/°C
SNR	78.61 ± 7.03 dB
Dynamic optical range	101.32 ± 12.41 dB

**Table 2:** Relationship between the number of cascaded probes, sampling rate, and total power consumption.

Number of Cascaded Probes	1	2	3	4	5	6	7	8
Number of valid channels	4	10	16	24	30	38	44	52
Sampling rate (Hz)	3000	1500	750	500	300	230	176	125
Total power consumption (mW)	176	253	302	344	385	434	476	512

**Table 3:** Comparative experimental outcomes of adaptive tuning.

	Measurement Site	Head	Upper Arm	Forearm	Chest	Neck	Thigh
Raw voltage (V)	Group I (With adaptive tuning)	2.450 ± 0.08	2.506 ± 0.11	2.496 ± 0.24	2.468 ± 0.07	2.504 ± 0.09	2.393 ± 0.18
	Group II (Without adaptive tuning)	0.998 ± 0.43	2.783 ± 0.61	3.134 ± 0.84	3.592 ± 0.52	2.311 ± 0.49	3.623 ± 0.95

**Table 4:** Stage-wise statistics of HbO concentration changes during the Valsalva maneuver.

		I	IIa	IIb	III	IV
HbO concentration changes (uM)	Subject 1	3.09 ± 0.79	−1.91 ± 1.25	0.58 ± 0.17	−2.06 ± 0.98	2.74 ± 1.32
	Subject 2	2.23 ± 0.82	−1.83 ± 0.62	0.67 ± 0.11	−2.35 ± 0.51	2.29 ± 0.89
	Subject 3	3.28 ± 3.64	−1.95 ± 0.82	−0.02 ± 0.17	−3.49 ± 1.80	4.66 ± 1.38

**Table 5:** Performance comparison with wearable NIRS systems.

Ref.	Maximum Sampling Rate (Hz)	Maximum Channel Count	Modular Cascading	Adaptive Tuning	Architecture	Power Consumption (mW)	DR (dB)	SNR (dB)
[13] 2017	100	-	Yes	No	CW-NIRS	≤400	>160	64
[37] 2018	-	128	No	No	CW-NIRS	-	-	32–70
[38] 2020	100	4	No	No	CW-NIRS	24		59
[16] 2020	48.825	256	No	Yes	CW-NIRS	-	-	-
[12] 2021	-	1728	Yes	No	CW-NIRS	-	106.6	-
[14] 2022	200	>2000	Yes	No	FD-NIRS	-	-	-
[10] 2025	1000	3	No	Yes	CW-NIRS	-	-	-
[17] 2025	16	8	No	No	CW-NIRS	-	-	58
[39] 2026	200	>64	Yes	No	CW-NIRS	295	-	-
[40] 2026	40	8	No	No	CW-NIRS	150	-	-
[41] 2026	1000	1	No	No	FD-NIRS	2750	55	-
[42] 2026	10	1	No	No	CW-NIRS	70–75	-	-
This Work	3000	52	Yes	Yes	CW-NIRS	176	101.32 ± 12.41	78.61 ± 7.03

## Data Availability

The raw data supporting the conclusions of this article will be made available by the authors upon reasonable request.
